# Endometrial Progesterone and Estrogen Receptors in Relation to Hormonal Levels in Women with Unexplained Recurrent Miscarriage

**DOI:** 10.1055/s-0043-1776030

**Published:** 2023-11-29

**Authors:** Ihab Adel Gomaa, Ahmed Sabry, Ihab Serag El-Din Allam, Sherif Ashoush, Ahmed Reda

**Affiliations:** 1 Ain Shams University Maternity Hospital, Abbaseya Square, Cairo, Egypt.

**Keywords:** Pregnancy loss, Receptor, Recurrent miscarriage, Estrogen, Progesterone

## Abstract

**Objective** Recurrent miscarriage has been linked to hormonal disturbance due to dysregulation of its receptors rather than to the availability of the hormone. We aimed to investigate endometrial expression of progesterone and estrogen receptors in relation to serum and endometrial hormonal levels in unexplained recurrent miscarriage.

**Methods** The present case control study included 20 cases with unexplained recurrent miscarriage and 20 parous women as controls. Ovulation was confirmed using an ovulation kit and 10 to 12 days after detecting the urinary luteinizing hormone surge, all women were subjected to a blood sample and to an endometrial biopsy. Progesterone and estrogen levels were measured in serum and in endometrial tissue and receptor concentrations were in the endometrial sample.

**Results** Women with recurrent miscarriage showed significantly lower concentration of receptors in both the cytoplasm and the nucleus of endometrial tissue compared with controls. The nuclear/cytoplasm ratio of progesterone receptor was significantly higher in cases compared with controls, implicating that recurrent miscarriage is probably linked to nongenomic activity of the hormone; this was also significant for estrogen receptor. Serum progesterone and estrogen hormonal levels were comparable between groups while both hormones were significantly reduced in the endometrium of recurrent miscarriage cases. Receptors significantly correlated with endometrial hormonal level but not to serum level.

**Conclusion** Recurrent miscarriage might be linked to reduced endometrial progesterone and estrogen receptors and appears to be more related to nongenomic activity of progesterone. Endometrial receptors expression correlates to tissue hormonal level rather than to serum hormonal level.

## Introduction

Recurrent miscarriage (RM) refers to “3 or more consecutive miscarriages.” 1 However, a diagnosis of RM could be considered after 2 consecutive miscarriages 2 3 on the basis that the incidence of a subsequent miscarriage does not significantly rise after the third compared to the second miscarriage. 2 Parental genetic abnormalities, uterine anatomical factors as well as antiphospholipid syndrome are agreed as direct causes for RM. Several other factors have been proposed but remain controversial and the existing evidence is either limited or inconclusive. 1 2 3 Cases presenting with RM are challenging because of the wide range of the proposed etiology and the diverse workup required for assessment; nevertheless, in > 50% of these, the cause remains unidentified.

Progesterone is one of the ovarian sex hormones which was demonstrated to play a crucial role for successful reproduction. 4 The interaction between progesterone and its receptors has been reported to initiate a paracrine effect preparing the endometrium for implantation as well as supporting the developing embryo and maintaining uterine quiescence throughout pregnancy. 5

Empirical use of progesterone has been proposed to address unexplained RM. 6 This practice was based on the assumption that insufficient hormonal level may be the cause of RM, but such management has yielded conflicting results. In a meta-analysis assessing the role of progesterone supplementation in prevention of RM, Haas et al. 7 concluded that a subsequent miscarriage may be reduced with progesterone therapy in women with unexplained RM. The PROMISE study, 8 on the other hand, demonstrated the ineffectiveness of progesterone in cases with RM. This might lead to hypothesize that the defect might be inadequate response of the receptors despite sufficient serum progesterone.

The objective of the present study is to investigate the endometrial expression of progesterone receptors (PR) and estrogen receptors (ER) in relation to serum and endometrial hormonal levels in women with unexplained RM.

## Methods

The present case control study was conducted between April 2018 and May 2020. The study protocol agreed with the Helsinki Declaration for ethical medical research and it was approved by the council of the obstetrics and gynecology department. After thorough explanation of the study, 40 women aged between 20 and 40 years old, having regular menstrual cycles for at least 3 months, signed an informed consent form and were enrolled in the present study. The sample size was calculated using G* power which showed that a sample of 20 cases per group with an allocation ratio of 1:1 is required to achieve an effect size of 1.09 with an α error = 0.05 and the study power was set at 0.9. The effect size was estimated from the results of a previous study. 9

The cases group included 20 women with unexplained RM defined as ≥ 3 prior spontaneous first trimester miscarriages with the last miscarriage within 6 months and prior investigations did not identify any cause for miscarriage. Prior workup in the course of management included normal hormonal levels, negative screening for antiphospholipid antibodies, exclusion of anatomical abnormalities by either 3D ultrasound, hysteroscopy or hysterosalpingography and normal karyotyping for both partners. Women with known genetic, anatomical, endocrine, autoimmune, or infectious disorders were excluded. The control group included 20 fertile women with at least 1 term birth presenting for unrelated conditions, women receiving hormonal treatment for any reason, and women with any known condition that may be related to miscarriage were excluded. The included women were instructed to avoid sexual intercourse or to use barrier contraception for the study duration (not to disturb an ongoing pregnancy during endometrial sampling) and to monitor their ovulation using a commercially available ovulation kits (Planny; DKT LLC) and they were scheduled for a return visit 10 to 12 days after detecting the urinary LH surge. They were subjected to an endometrial biopsy obtained using a pipelle curette and it was frozen in liquid nitrogen until the time of analysis. On the same day, a 5 cc blood sample was withdrawn and was left to clot in room temperature, then it was centrifuged and the serum was separated and frozen until the time of assay.

## Assessment of ER and PR in the Endometrial Sample

Total receptors concentration was assessed in the endometrial sample using the technique described in a previous study. 9 The tissue was homogenated at 4°C then it was centrifuged for 60 minutes to obtain the supernatant (cytosol fraction). The ‘raw’ button was dissolved in buffer and incubated for 1 hour, during which the pellet was dissolved every 15 minutes. The solubilized proteins were obtained by centrifugation (nucleosol fraction). The ER was measured incubating the cytosol and the nucleosol (200 ml) in several concentrations (0.25–5.0 mM) of 3H-estradiol during 18 to 20 hours at 4°C. The nonspecific union was analyzed using diethylstillbestrol in excess (200). The PR was determined using 3H-ORG-2058 (0.25–10 mM) for 18 to 20 hours at 4°C. Both receptors were quantified using a Scatchard analysis and the proteins values were determined in the cytosol and in the nucleosol using the Lowry method. The sensitivity limits of this method were 1.0 to 75 and 1.0 to 50 fmol/mg-protein for ER and PR, respectively. The intra- and interassay relative standard deviation (SD) for the ER and PR were < 6%.

## Assessment of Endometrium and Serum Hormonal Level

Estradiol and progesterone concentrations in the collected endometrial biopsy and serum were measured by a high-performance liquid chromatography method performed with an Agilent Series 1050 quaternary gradient pump, Series 1050 auto sampler, Series 1050 UV Vis detector (Agilent co., Germany), and HPLC 2D Chemstation software (Hewlett-Packard, Les Ulis, France) following a previously described technique. 10

## Results

**Fig. 1:**
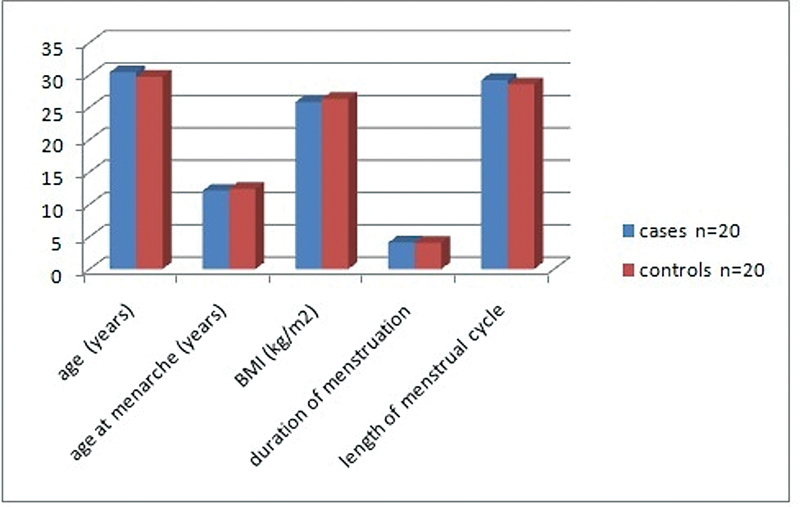
Baseline demographics and background history.

The cases group included 12 women (60%) with a history of prior 3 miscarriages and 8 women (40%) with 4 previous miscarriages; background history was comparable between groups ( Fig. 1 ).

**Fig. 2:**
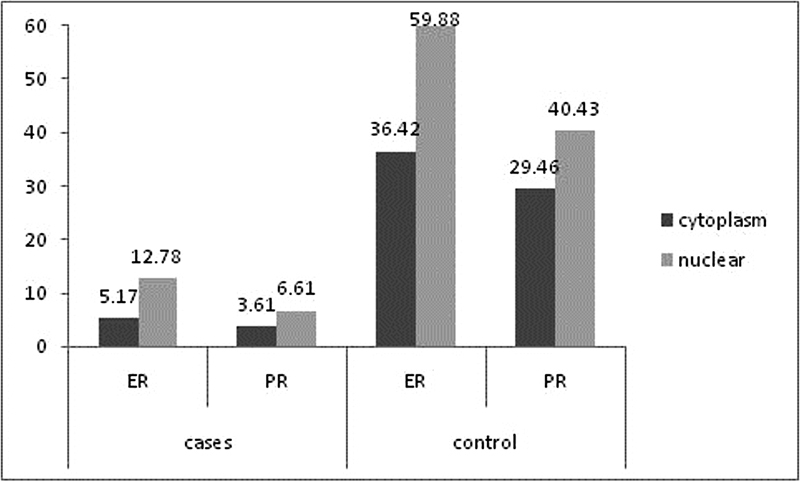
Comparison between cytoplasm and nuclear concentration of progesterone and estrogen receptors among cases and controls.

The concentration of PR and ER was measured in the cytoplasm as well as in salt extracted nucleus. Compared with controls, women with RM showed a significantly lower concentration of PR in the cytoplasm (3.61 ± 1.52 versus 29.46 ± 10.56 fmol/mg protein; p < 0.001) and in the nucleus (6.61 ± 1.63 versus 40.43 ± 9.35 fmol/mg protein; *p* < 0.001). Estrogen receptor concentration was also significantly reduced in the cytoplasm (5.17 ± 1.9 versus 36.42 ± 12.8 fmol/mg protein; *p* < 0.001) and in the nucleus (12.78 ± 4.08 versus 59.88 ± 19.69 fmol/mg protein; *p* < 0.001) among women with RM compared with controls. In both groups, PR and ER were more expressed in the nucleus more than in the cytoplasm ( Fig. 2 ).

**Fig. 3:**
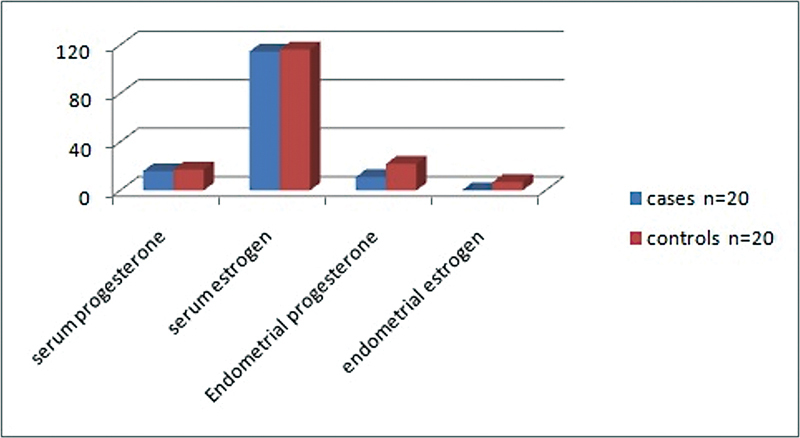
Comparison between serum and endometrial hormonal levels among cases and controls.

It was also noticed that the receptor concentration is more reduced in the cytoplasmic compartment. The nuclear/cytoplasm ratio of PR was significantly higher in cases (2.15 ± 0.92) compared with controls (1.54 ± 0.65; *p* = 0.02); this ratio was also significant for ER (2.56 ± 0.62 in cases versus 1.78 ± 0.68 in controls; *p* = 0.001). Regarding the PR/ER ratio, neither the cytoplasm nor the nuclear compartment was significantly different between cases and controls ( *p* > 0.05). Progesterone and estrogen serum levels were comparable between groups while both hormones were significantly reduced in the endometrium of cases with RM ( Fig. 3 ). The mean of serum progesterone was 15.79 ± 2.94 ng/ml in cases compared with 17.22 ± 3.02 ng/ml in controls while serum estrogen was 114.56 ± 7.35 pg/ml in cases compared with 116.1 ± 7.88 pg/ml in controls; this difference was statistically not significant ( *p* > 0.05). Endometrial progesterone was 10.86 ± 3.11 versus 21.85 ± 3.3 ng/ml among cases and controls respectively ( *p* < 0.001), and endometrial estrogen level was 0.0437 ± 0.03 compared with 6.77 ± 3.55 ng/ml in cases and controls, respectively ( *p* < 0.001). Correlations between receptors and hormonal levels are demonstrated in Charts 1 and 2 . Both receptors were positively correlated with the endometrial level of its hormone and inversely related to each other, and these correlations were statistically significant. Both receptors significantly correlated positively with the endometrial level of its hormone while correlation to the serum level was not significant. These observations were found in cases as well as in controls.

**Chart 1:** Correlations between receptor concentrations and hormonal levels among cases

	PR (cytoplasm)	PR (nuclear)	ER (cytoplasm)	ER (nuclear)	Serum progesterone	Endometrial progesterone	Serum estrogen	Endometrial estrogen
PR (cytoplasm)	—	0.373 (0.11)	- 0.924 ^*^ (< 0.001)	- 0.874 ^*^ (< 0.001)	0.326 (0.16)	0.791 ^*^ (< 0.001)	- 0.312 (0.18)	- 0.692 ^*^ (0.001)
PR (nuclear)	0.373 (0.11)	—	- 0.46 ^*^ (0.04)	- 0.359 (0.12)	0.085 (0.72)	0.412 (0.07)	- 0.096 (0.69)	- 0.276 (0.24)
ER (cytoplasm)	- 0.924 ^*^ (< 0.001)	- 0.46 ^*^ (0.04)	—	0.838 ^*^ (< 0.001)	- 0.218 (0.36)	- 0.728 ^*^ (<0.001)	0.195 (0.41)	0.804 ^*^ (< 0.001)
ER (nuclear)	- 0.874 ^*^ (< 0.001)	- 0.359 (0.12)	0.838 ^*^ (< 0.001)	—	- 0.396 (0.08)	- 0.64 ^*^ (0.002)	0.37 (0.11)	0.599 ^*^ (0.005)
Serum progesterone	0.326 (0.16)	0.085 (0.72)	- 0.218 (0.36)	- 0.396 (0.08)	—	0.377 (0.1)	- 0.344 (0.14)	- 0.214 (0.36)
Endometrial progesterone	0.791 ^*^ (< 0.001)	0.412 (0.07)	- 0.728 ^*^ (< 0.001)	- 0.64 ^*^ (0.002)	0.377 (0.1)	—	- 0.367 (0.11)	- 0.55 ^*^ (0.01)
Serum estrogen	- 0.312 (0.18)	- 0.096 (0.69)	0.195 (0.41)	0.37 (0.11)	- 0.344 (0.14)	- 0.367 (0.11)	—	0.319 (0.17)
Endometrial estrogen	- 0.692 ^*^ (0.001)	- 0.276 (0.24)	0.804 ^*^ (< 0.001)	0.599 ^*^ (0.005)	- 0.214 (0.36)	- 0.55 ^*^ (0.01)	0.319 (0.17)	—

Abbreviations: ER, estrogen receptor; PR, progesterone receptor.

Analysis done using the Pearson correlation test; data presented as r value ( *p* -value),

*statistically significant

**Chart 2:** Correlations between receptor concentrations and hormonal levels among controls

	PR (cytoplasm)	PR (nuclear)	ER (cytoplasm)	ER (nuclear)	Serum progesterone	Endometrial progesterone	Serum estrogen	Endometrial estrogen
PR (cytoplasm)	—	0.185 (0.43)	- 0.926 ^*^ (< 0.001)	- 0.742 ^*^ (< 0.001)	- 0.182 (0.44)	0.639 ^*^ (0.002)	0.144 (0.54)	- 0.536 ^*^ (0.02)
PR (nuclear)	0.185 (0.43)	—	- 0.054 (0.82)	- 0.038 (0.87)	0.133 (0.58)	0.141 (0.55)	0.249 (0.29)	- 0.537 ^*^ (0.02)
ER (cytoplasm)	- 0.926 ^*^ (< 0.001)	- 0.054 (0.82)	—	0.613 ^*^ (0.004)	0.126 (0.6)	- 0.526 ^*^ (0.02)	0.125 (0.6)	0.396 (0.08)
ER (nuclear)	- 0.742 ^*^ (< 0.001)	- 0.038 (0.87)	0.613 ^*^ (0.004)	—	0.332 (0.15)	- 0.577 ^*^ (0.008)	0.085 (0.72)	0.572 ^*^ (0.008)
Serum progesterone	- 0.182 (0.44)	0.133 (0.58)	0.126 (0.6)	0.332 (0.15)	—	0.035 (0.88)	- 0.094 (0.69)	0.061 (0.8)
Endometrial progesterone	0.639 ^*^ (0.002)	0.141 (0.55)	- 0.526 ^*^ (0.02)	- 0.577 ^*^ (0.008)	0.035 (0.88)	—	- 0.148 (0.44)	- 0.437 (0.05)
Serum estrogen	0.144 (0.54)	0.249 (0.29)	0.125 (0.6)	0.085 (0.72)	- 0.094 (0.69)	- 0.148 (0.44)	—	- 0.258 (0.27)
Endometrial estrogen	- 0.536 ^*^ (0.02)	- 0.537 ^*^ (0.02)	0.396 (0.08)	0.572 ^*^ (0.008)	0.061 (0.8)	- 0.437 (0.05)	- 0.258 (0.27)	—

Abbreviations: ER, estrogen receptor; PR, progesterone receptor.

Analysis done using the Pearson correlation test; data presented as r value ( *p* -value),

*statistically significant

## Discussion

The present case control study compared 20 women with unexplained RM with 20 parous controls. Compared with controls, women with RM showed a significantly lower concentration of receptors in the cytoplasm as well as in the nuclear compartments of endometrial tissue. The nuclear/cytoplasm ratio of both PR and ER was significantly higher in cases compared with controls, which implicates that RM seems linked to nongenomic activity of the hormones. Serum hormonal levels were comparable between groups while both hormones were significantly reduced in the endometrium of cases with RM and receptors significantly correlated positively with the endometrial hormonal level but not with serum level.

Progesterone receptors are expressed in 2 isoforms; PR-A and PR-B, with other PR isoforms of uncertain physiological relevance. Both isoforms are expressed during the proliferative phase and increase with rising estrogen level, then PR-B remains constant while PR-A declines in the late secretory phase. 11 A defect in progesterone function has been attributed to dysregulation of PR rather than the availability of the hormone and disturbed receptor expression has been linked to pregnancy loss, although the exact mechanism is poorly understood. 12 Dysregulation of PR may be associated with reduced receptor expression or linked to genetic polymorphisms of PR affecting its response to progesterone. 13 14 Furthermore, RM may be linked to progesterone resistance as a result of epigenetic modification; this has been reported in a baboon model of endometriosis. 15

Estrogen hormone has also been linked to miscarriage. Estrogen binds to its receptors to stimulate uterine cells to express PR thus promoting the endometrial response to progesterone. 11 Estrogen receptors are expressed in 2 isoforms, alpha and beta, which promote endometrial priming for pregnancy. During the proliferative phase, ERα expression is at its greatest, then it declines in the secretory phase. 16 A significant reduction of ER expression in decidua has been reported in women with early spontaneous miscarriage compared with women presenting for elective termination of pregnancy 17 and ER dysregulation has also been demonstrated to alter the expression of PR. 15

Isoforms expression of both PR and ER can be assessed using biochemical, real-time RT-PCR or immunohistochemical methods. The common sequence of the isoforms limits the quantification of individual receptor isoform. 11 It has been demonstrated that there is unequal efficacy of the different antibodies used for immunohistochemical tests in recognizing receptor isoforms. 18 The present study evaluated the total concentration of the receptors and did not account for the different receptor isoforms due to technical limitations in accurately quantifying separate isoforms and derived by the consideration that the endometrial response to hormones represents the combined activities of its isoforms.

The present study shows that compared with fertile women, individuals with unexplained RM had significantly lower PR and ER concentrations in the nuclear and cytoplasmic compartment. In agreement with our findings, Rahnama et al. 19 demonstrated that PR expression was significantly lower in women with RM and the authors suggested that this difference may be linked to RM. Also in line with the present results, Salazar et al. 20 reported a significantly lower PR and ER in cases with RM compared with fertile women.

In another study, Carranza-Lira et al. ^9^ reported that cytoplasmic PR was significantly lower in women with RM but, contrary to our results, that nuclear PR was significantly lower in controls. However, that study agreed with the present results in that ER in cytoplasmic and nuclear compartment were lower in women with RM but it did not reach significance. The differences from the present results may be attributed to the small sample of that study as it only included 5 women with RM and 6 controls.

Also in agreement with the present results, Liang et al. 21 studied the expression of PR in decidual tissue of women with unexplained RM in comparison with normal pregnant women subjected to induced abortion using immunohistochemistry and they reported significant down regulation of PR expression in the RM group (0.1632 ± 0.007 versus 0.2122 ± 0.01; *p* = 0.0003).

As noted from previous studies, 11 16 there is a physiological variation throughout the menstrual cycle in endometrial expression of both PR and ER, but the present study evaluated the status of the receptors in the midluteal phase which represents the most relevant period for establishing and maintenance of pregnancy.

Currently, progesterone is demonstrated to function through a genomic activity mediated via the nuclear receptors and a nongenomic activity mediated via the extranuclear receptors. 22 Contrary to our results, nuclear receptors are hypothesized to be the primary mechanism for progesterone action in the human female reproductive system. 11 The present study demonstrates that the cytoplasmic fraction of PR is significantly more reduced than the nuclear fraction in women with RM, thus implicating the nongenomic activity in the pathophysiology of RM. Further studies are needed to explore this observation.

The physiology of female reproduction is regulated by ovarian steroids which coordinate to produce a favorable environment required for embryo implantation and progression of pregnancy. This is established by modulation of maternal immune system and although no clear mechanism has been reported implicating progesterone in this action, 11 it has been reported that decidualization of the endometrium depends on adequate progesterone level together with endometrial expression of PR to mediate its effect. 23 A decline in serum progesterone and estrogen has also been reported in women with early spontaneous miscarriage compared with women presenting for elective termination of pregnancy. 24

Currently, there is no agreement on a cutoff for serum progesterone level that best defines ovulatory cycles or may predict pregnancy outcome. Several levels for midluteal progesterone have been suggested to confirm ovulation. Only two retrospective studies reported on hormonal levels in cycles ending up with pregnancy. 17 25 Takaya et al. 17 reported that a minimum of 5.6 ng/ml serum progesterone and 70.2 pg/ml estrogen is required to achieve pregnancy. A higher value was reported in an earlier study in which Sallam et al. 25 reported that a minimum of 10.83 ng/ml is required to achieve pregnancy; however, they assessed women who underwent induction of ovulation with human menopausal gonadotrophin which is known to increase progesterone levels.

The mean serum hormonal level in the present population was comparable to that of parous women and it was above the previously demonstrated cutoffs required for favorable pregnancy outcome. The adequate serum hormones in the current population rules out serum progesterone decline as the sole cause of RM outside the context of luteal phase deficiency. In addition, it might provide explanation for the heterogeneous evidence regarding empiric progesterone supplementations in women with RM.

On the other hand, tissue concentration seems to have a role in the pathophysiology of RM according to the present results, which showed that progesterone and estrogen concentrations were significantly lower in the endometrial sample of women with unexplained RM compared with fertile women. Variation in blood concentration of sex steroids has been demonstrated to influence the endometrial expression of PR and ER 26 and it was suggested that both receptors change in line to blood or endometrial changes of sex steroids. 27 28 This in part agrees with the present results as both PR and ER were found to significantly correlate with endometrial hormonal concentration but not to serum levels.

In agreement with our results, Li et al. 29 reported no significant difference between serum estrogen in RM compared with normal fertile women. Also, Salazar et al. 20 reported a significant reduction in endometrial progesterone concentration in RM women but in disagreement with our results, they reported a comparable estrogen concentration and significantly lower serum progesterone.

A limitation of the present study is the evaluation of the total concentration of the receptor without assessing if there is a difference in different isoforms expression, but this point can be argued by the technical difficulty in quantifying receptor isoforms and also by the consideration that progesterone action represents the combined activities of the isoforms. Another limitation is the retrospective nature of the study. A strength of the present study is the evaluation of the hormonal function as a unit with assessment of the hormones at serum and tissue combined with receptor expression. This highlighted a subgroup with a normal serum hormonal level but with a suboptimal response related to aberrant receptor expression, also explaining in part the heterogeneous response to empirical progesterone supplementations in RM excluding a subgroup which obviously will not benefit from such treatment.

## Conclusion

Recurrent miscarriage might be linked to reduced endometrial progesterone and estrogen receptors and appears to be more related to nongenomic activity of progesterone. Endometrial receptors expression correlates to tissue hormonal level rather than to serum hormonal level.

## References

[1] Royal College of Obstetricians and gynecologists.The investigation and treatment of couples with recurrent first-trimester and second-trimester miscarriageGreen top guideline no. 17 (2011

[2] American College of Obstetricians and gynecologists. ACOG practice bulletin. management of recurrent pregnancy loss Int J Gynaecol Obstet 2002 78 02 179 190 10.1016/s0020-7292(02)00197-2 12360906

[3] ESHRE Guideline Group on RPL Bender Atik R Christiansen O B Elson J Kolte A M Lewis S Middledorp S ESHRE guideline: recurrent pregnancy loss Hum Reprod Open 2018 2018 02 hoy004 10.1093/hropen/hoy004 31486805 PMC6276652

[4] Di Renzo G C Giardina I Clerici G Brillo E Gerli S Progesterone in normal and pathological pregnancy Horm Mol Biol Clin Investig 2016 27 01 35 48 10.1515/hmbci-2016-0038 27662646

[5] Wetendorf M DeMayo F J The progesterone receptor regulates implantation, decidualization, and glandular development via a complex paracrine signaling network Mol Cell Endocrinol 2012 357(1-2):108 118 10.1016/j.mce.2011.10.028 22115959 PMC3443857

[6] Check J H Cohen R The role of progesterone and the progesterone receptor in human reproduction and cancer Expert Rev Endocrinol Metab 2013 8 05 469 484 10.1586/17446651.2013.827380 30754194

[7] Haas D M Hathaway T J Ramsey P S Progestogen for preventing miscarriage in women with recurrent miscarriage of unclear etiology Cochrane Database Syst Rev 2019 2019 11 CD003511 10.1002/14651858.CD003511.pub5 31745982 PMC6953238

[8] Coomarasamy A Williams H Truchanowicz E Seed P T Small R Quenby S PROMISE: first-trimester progesterone therapy in women with a history of unexplained recurrent miscarriages - a randomised, double-blind, placebo-controlled, international multicentre trial and economic evaluation Health Technol Assess 2016 20 41 1 92 10.3310/hta20410 PMC4904188 27225013

[9] Carranza-Lira S Blanquet J Tserotas K Calzada L Endometrial progesterone and estradiol receptors in patients with recurrent early pregnancy loss of unknown etiology–preliminary report Med Sci Monit 2000 6 04 759 762 11208405

[10] Häkkinen M R Heinosalo T Saarinen N Linnanen T Voutilainen R Lakka T Analysis by LC-MS/MS of endogenous steroids from human serum, plasma, endometrium and endometriotic tissue J Pharm Biomed Anal 2018 152 165 172 10.1016/j.jpba.2018.01.034 29414008

[11] Patel B Elguero S Thakore S Dahoud W Bedaiwy M Mesiano S Role of nuclear progesterone receptor isoforms in uterine pathophysiology Hum Reprod Update 2015 21 02 155 173 10.1093/humupd/dmu056 25406186 PMC4366574

[12] Agrawal V Jaiswal M K Jaiswal Y K Lipopolysaccharide-induced modulation in the expression of progesterone receptor and estradiol receptor leads to early pregnancy loss in mouse Zygote 2012 10.1017/S0967199412000330PubMed 22809764

[13] Bahia W Finan R R Al-Mutawa M Haddad A Soua A Janhani F Genetic variation in the progesterone receptor gene and susceptibility to recurrent pregnancy loss: a case-control study BJOG 2018 125 06 729 735 10.1111/1471-0528.14949 28972310

[14] Razdaibiedina A Khobzey M Tkachenko V Vorobiova I Effects of Single-Nucleotide Polymorphisms in Cytokine, Toll-Like Receptor, and Progesterone Receptor Genes on Risk of Miscarriage Hindawi Obstet Gynecol International 2018 2018 10.1155/2018/9272749PubMed PMC6079348 30116270

[15] Fazleabas A T Progesterone resistance in a baboon model of endometriosis Semin Reprod Med 2010 28 01 75 80 10.1055/s-0029-1242997 20104431

[16] Gibson D A Esnal-Zufiaurre A Bajo-Santos C Collins F Critchley H OD Saunders P TK Profiling the expression and function of oestrogen receptor isoform ER46 in human endometrial tissues and uterine natural killer cells Hum Reprod 2020 35 03 641 651 10.1093/humrep/dez306 32108901 PMC7105323

[17] Takaya Y Matsubayashi H Kitaya K Nishiyama R Yamaguchi K Takeuchi T Minimum values for midluteal plasma progesterone and estradiol concentrations in patients who achieved pregnancy with timed intercourse or intrauterine insemination without a human menopausal gonadotropin BMC Res Notes 2018 11 01 61 10.1186/s13104-018-3188-x 29357944 PMC5778625

[18] Mote P A Johnston J F Manninen T Tuohimaa P Clarke C L Detection of progesterone receptor forms A and B by immunohistochemical analysis J Clin Pathol 2001 54 08 624 630 11477119 10.1136/jcp.54.8.624 PMC1731503

[19] Rahnama R Rafiee M Fouladi S Akbari-Fakhrabadi M Mehrabian F Rezaei A Gene expression analysis of membrane progesterone receptors in women with recurrent spontaneous abortion: a case control study BMC Res Notes 2019 12 01 790 10.1186/s13104-019-4787-x 31801604 PMC6894300

[20] Salazar E L Calzada L The role of progesterone in endometrial estradiol- and progesterone-receptor synthesis in women with menstrual disorders and habitual abortion Gynecol Endocrinol 2007 23 04 222 225 10.1080/09513590701254030 17505942

[21] Liang Q Tong L Xiang L Shen S Pan C Liu C Correlations of the expression of γδ T cells and their co-stimulatory molecules TIGIT, PD-1, ICOS and BTLA with PR and PIBF in the peripheral blood and decidual tissues of women with unexplained recurrent spontaneous abortion Clin Exp Immunol 2021 203 01 55 65 10.1111/cei.13534 33017473 PMC7744496

[22] Marquardt R M Kim T H Shin J H Jeong J W Progesterone and Estrogen Signaling in the Endometrium: What Goes Wrong in Endometriosis? Int J Mol Sci 2019 20 15 3822 10.3390/ijms2015382 31387263 PMC6695957

[23] Mulac-Jericevic B Conneely O M Reproductive tissue selective actions of progesterone receptors Reproduction 2004 128 02 139 146 15280552 10.1530/rep.1.00189

[24] Verma P Verma R Nair R R Budhwar S Khanna A Agrawal N R Altered crosstalk of estradiol and progesterone with Myeloid-derived suppressor cells and Th1/Th2 cytokines in early miscarriage is associated with early breakdown of maternal-fetal tolerance Am J Reprod Immunol 2019 81 02 e13081 10.1111/aji.13081 30589483

[25] Sallam H N Sallam A Ezzeldin F Agamia A F Abou-Ali A Reference values for the midluteal plasma progesterone concentration: evidence from human menopausal gonadotropin-stimulated pregnancy cycles Fertil Steril 1999 71 04 711 714 10202883 10.1016/s0015-0282(98)00531-7

[26] Conneely O M Mulac-Jericevic B Lydon J P De Mayo F J Reproductive functions of the progesterone receptor isoforms: lessons from knock-out mice Mol Cell Endocrinol 2001 179(1-2):97 103 10.1016/s0303-7207(01)00465-8 11420134

[27] Sabbir M G Progesterone induced Warburg effect in HEK293 cells is associated with post-translational modifications and proteasomal degradation of progesterone receptor membrane component 1 J Steroid Biochem Mol Biol 2019 191 105376 10.1016/j.jsbmb.2019.105376 31067491

[28] Diep C H Ahrendt H Lange C A Progesterone induces progesterone receptor gene (PGR) expression via rapid activation of protein kinase pathways required for cooperative estrogen receptor alpha (ER) and progesterone receptor (PR) genomic action at ER/PR target genes Steroids 2016 114 48 58 10.1016/j.steroids.2016.09.004 27641443 PMC5068826

[29] Li T C Spuijbroek M D Tuckerman E Anstie B Loxley M Laird S Endocrinological and endometrial factors in recurrent miscarriage BJOG 2000 107 12 1471 1479 11192102 10.1111/j.1471-0528.2000.tb11670.x

